# Optimizing COVID-19 treatment in immunocompromised patients: early combination therapy with remdesivir, nirmatrelvir/ritonavir and sotrovimab

**DOI:** 10.1186/s12985-023-02269-8

**Published:** 2023-12-15

**Authors:** Ivan Gentile, Maria Foggia, Maria Silvitelli, Alessia Sardanelli, Letizia Cattaneo, Giulio Viceconte

**Affiliations:** Department of Clinical Medicine and Surgery, Section of Infectious Diseases, University of Naples “AOU Federico II”, Via Sergio Pansini,5, Naples, 80131 Italy

**Keywords:** COVID-19, Immunocompromised, Sotrovimab, Remdesivir, Nirmatrelvir

## Abstract

**Background:**

Morbidity and mortality are higher in immunocompromised patients affected by COVID-19 than in the general population. Some authors have successfully used antiviral combination, but never in the early phase of the infection.

**Methods:**

We conducted a retrospective cohort study to determine the efficacy and safety of the combination of two antivirals, with and without a monoclonal antibody (mAb), in both the early (within 10 days of symptoms) and in a later phase (after 10 days) of SARS-CoV-2 infection in immunocompromised patients admitted to our Facility.

**Results:**

We treated 11 patients (seven in an early phase and four in a late phase of COVID-19) with 10 days of intravenous remdesivir plus five days of oral nirmatelvir/ritonavir, also combined with sotrovimab in 10/11 cases. Notably, all the “early” patients reached virological clearance at day 30 from the end of the therapy and were alive and well at follow-up, whereas the corresponding numbers in the “late” patients were 50% and 75%. Patients in the “late” group more frequently needed oxygen supplementation (p = 0.015) and steroid therapy (p = 0.045) during admission and reached higher COVID-19 severity (p = 0.017).

**Discussion:**

The combination of antiviral and sotrovimab in the early phase of COVID-19 is well tolerated by immunocompromised patients and is associated with 100% of virological clearance. Patients treated later have lower response rates and higher disease severity, but whether therapy plays a causative role in such findings has yet to be determined.

## Background

Immunocompromised patients are at a high risk of progression and death from COVID-19 [1]. Moreover, patients with impaired humoral immunity have the highest risk of prolonged infection, viral and clinical rebound, and the highest mortality rate among immunocompromised patients [1]. Various conditions are associated with impaired humoral immunity, namely B-cell depletion due to primary conditions (X-linked agammaglobulinaemia, common variable immunodeficiency, other types of primary hypogammaglobinaemia) or secondary causes (anti-CD20 treatment in the previous year; chronic lymphoblastic leukemia, non-Hodgkin lymphoma, multiple myeloma) [2]. Such patients can develop “persistent inflammatory seronegative COVID” which is a new definition of COVID-19, associated with prolonged or relapsing symptoms and persistent SARS-CoV-2 RT-PCR on respiratory samples in the absence of serological response to the infection after 14 days [2]. In such cases, there is no established therapy that can both reduce risk of progression and the time to virological clearance. Some authors have described the use of antiviral combinations with or without mAbs, but only in prolonged/relapsing COVID-19 and never in the early phase of the infection [3–6].

Recently, Tam et al. published the results of a phase 2 open label randomized trial that demonstrated that early treatment (within three days) with interferon beta-1b and remdesivir was safe and better than remdesivir only in alleviating symptoms, shortening viral shedding and hospitalization with earlier seropositivity in high-risk COVID-19 patients [7].

In April 2023, we started to use a combination of two antivirals with mAbs both in prolonged/relapsing COVID-19 and as early treatment (within 10 days from symptom onset) in immunocompromised patients at a high risk of developing prolonged/relapsing infections.

## Methods

Retrospective cohort studies including all patients with non-critical, mild-to-severe symptomatic COVID-19, admitted to the Infectious Disease Ward of Federico II University Hospital from April to June 2023 and treated with at least two antivirals with or without an mAb active or presumably active against the circulating variant during the study.

The off-label administration of the drugs was approved case-by-case by the Italian regulatory agency on drugs (Agenzia Italiana del Farmaco- AIFA), the Chief Medical Officer of the Federico II University Hospital and the Chief of the Hospital Pharmacy, as required by the Italian law and by the Hospital’s regulations.

COVID-19 was diagnosed based on the detection of SARS-Cov-2 RNA on nasopharyngeal swab (NFS) with real-time PCR, together with typical symptoms. Patients were monitored for a median of 44 (IQR 10–92) days after the administration of the combination therapy with clinical supervision and NFS was collected every five days until a negative result was obtained. The outcomes were: (i) virological clearance, defined as negative SARS-CoV-2 PCR in NFS at day 30 post-treatment; (ii) clinical response, defined as being well, and with negative NFS at the last follow-up.

Statistical analysis was performed using SPSS version 27 (SPSS Inc. Chicago, IL). Continuous variables were reported as median and interquartile range, and categorical variables as frequencies and percentages. Categorial variables were compared with the Chi-squared and Fisher’s exact tests as appropriate. Continuous variables were compared with the Mann-Whitney U test. A significance level of 0.05 was set for the interpretation of the results. Multivariable analysis was not performed due to a limited number of events.

## Results

During the observation period, we treated 11 immunocompromised patients with 10 days of intravenous remdesivir plus 5 days of oral nirmatrelvir/ritonavir (N/r), combined with sotrovimab in 10/11 cases on the fifth day after remdesivir initiation.

Seven of 11 patients received combination therapy within 10 days (a median of 5 days, IQR 3–9) from COVID-19 symptoms onset (“early treatment group”), while 4/11 after a median of 79 (IQR 48–112) days (“late treatment group”). In detail, among the “early treatment group”, two patients started treatment at 3, one at 4, one at 5, two at 9 and one at 10 days from symptom onset.

All patients had hematologic malignancies except one who was affected by Good’s syndrome associated with malignant thymoma and another receiving rituximab for multiple sclerosis. Demographic and clinical characteristics of the patients are shown in Table 1.

**Table 1:** Demographic and clinical characteristics

	Overall N = 11	Early combination treatment N = 7	Late combination treatment N = 4	p-value
Age, years, median (IQR)	51 (36–57)	52 (35–56)	63 (42–77)	0.667
Females, n (%)	5 (45.5)	3 (43)	2 (50)	0.652
Charlson Comorbidity Index, median (IQR)	0.5 (0-1.25)	0 (0–1)	2 (0.25-3)	0.517
Vaccinated, n (%)	8 (72)	5 (71)	3 (75)	0.721
SARS-CoV-2 vaccine doses, median (IQR)	3 (3)	3 (3)	3 (3)	1
Hematologic malignancy				
Acute myeloid leukemia	3 (27)	3 (42)	0 (0)	0.3
Non-Hodgkin lymphoma	4 (36)	2 (28)	2 (50)	0.646
Chronic lymphatic leukemia	1 (9)	1 (14)	0 (0)	0.308
Multiple myeloma	1 (9)	0 (0)	1 (25)	0.462
Other immunodeficiencies, n (%)	2 (18)	1 (14)	1 (25)	0.538
Use of anti-CD20, n (%)	2 (18)	0 (0)	2 (50)	0.109
Deaths, n (%)	0 (0)	0 (0)	0 (0)	
Duration of hospital stay, days, median (IQR)	14 (12.5–23)	13 (11.5–16.5)	21 (12.5–38)	0.2
Early COVID-19 therapy, n (%)	1 (9)	0 (0)	1 (25)	0.364
Preventive TIX-CIL, n (%)	1 (9)	1 (14)	0 (0)	0.325
Previous therapy for COVID-19, n (%)	3 (27)	0 (0)	3 (75)	0.8
Days from COVID-19 symptoms to combination therapy, median (IQR)	9 (3.75-48)	5 (3–9)	79 (48–112)	0.017
Remdesivir 10 d + N/r, n (%)	11	7	4	
Remdesivir 10 d + N/r + Sotrovimab, n (%)	10	6	4	
SARS-CoV-2 infection duration from the end of therapy, days, median (IQR)	10 (7-22.5)	9 (8–17)	30 (4.7–55)	0.55
Negative NFS at day 14n (%)	4 (36.4)	4 (57)	0 (0)	0.106
Negative NFS at day 30, n (%)	9 (81)	7 (100)	2 (50)	0.109
Aive and well at FU, n (%)	10 (90)	7 (100)	3 (75)	0.364
Days of follow up, median (IQR)	44 (10–92)	40 (1–81)	65 (11–108)	0.183
Use of immunomodulatory drug for COVID-19, n (%)	2 (18)	0 (0)	2 (50)	0.109
COVID-19 steroid treatment, n (%)	6 (54)	2 (29)	4 (100)	0.045
Worst WHO severity grade, median (IQR)	3 (3-4.2)	3 (3)	4.5 (4–5)	0.017
Need for O2, n (%)	5 (45.5)	1 (14)	4 (100)	0.015
ADR, n (%)	1 (9)	1 (14)	0	0.636
Lowest lymphocyte value, cells/mm^3^, median (IQR)	0.39 (0.15–0.9)	0.68 (0.2–1.2)	0.16 (0.07–0.32)	0.067
Highest CRP value, mg/dL, median (IQR)	3.8 (0.3–15)	2.3 (0.3-5)	17 (8-23.4)	0.117
Highest LDH value, IU/mL, median (IQR)	287 (231–446)	251 (225–436)	488 (297–708)	0.267
Highest ferritin value, median (IQR)	495 (302–1635)	424 (253–907)	2000 (542–2000)	0.286
Highest D-dimer, median (IQR)	863 (571–1580)	620 (571–1658)	1422 (1084–1931)	0.383

*TIX-CIL: tixagevimab-cilgavimab; N/r: nirmatrelvir/ritonavir; NFS: nasopharyngeal swab; FU: follow-up; WHO: World Health Organization; ADR: adverse drug reaction; CRP: C-reactive protein*

Patients in the late treatment group suffered from a prolonged course of COVID-19, with a median of 79 (IQR 48–112) days from COVID-19 symptoms before initiating combination therapy. During the latter all patients reported an history of several clinical relapse of symptoms and they have documented detectable SARS-CoV-2 on multiple NFSs, with no evidence of negative RT-PCR assay during the course of the disease.

Only one patient in the late treatment group had received an early therapy for COVID-19 in the first 7 days of symptoms: 3 days of remdesivir two days after the onset of COVID-19 symptoms, and 30 days before receiving the combination therapy. Conversely, 3 of 4 patients in the late treatment group affected by relapsing COVID-19 had clinical and radiological confirmed SARS-CoV-2 pneumonia and was treated with intravenous remdesivir, together with dexamethasone and oxygen, for 10 days, namely, a median of 35 days (IQR 8–40) after symptom onset. This treatment resulted in an initial clinical response (discontinuation of the reduction in oxygen symptoms), without however obtaining complete viral clearance. Upon clinical relapse the patients were treated with combination therapy for a median of 32 days (IQR 32–50) after the first treatment. Only one patient (in the early group) had an adverse drug reaction (symptomatic bradycardia) which led to remdesivir discontinuation after 8 days of therapy.

As shown in Table 1, the two groups of patients did not differ in terms of baseline demographic and clinical conditions. Notably, all the “early” patients reached virological clearance at day 30 from the end of treatment and were alive and well at follow-up. In contrast, corresponding numbers in the “late” patients were 50% and 75%. One patient of this group died with persistence of infection at follow-up. In detail, in the “early treated” group, 4 patients attained viral clearance within 14 days from the end of the combination therapy and the remaining 3 within 30 days. Conversely, no patients in the “late treatment” group reached viral clearance within 14 days, two had negative NFS within 30 days, one after 50 days post-therapy and one died of SARS-Cov-2 infection 57 days after the end of combination therapy. Notably, patients in the late group more frequently needed oxygen supplementation (p = 0.015) and steroid therapy (p = 0.045) during admission. Moreover, they had a higher World Health Organization (WHO) COVID-19 severity score (p = 0.017) [8] compared to early treated patients, while no significant differences were found in terms of laboratory values associated with COVID severity (lymphocyte count, C-reactive protein, LDH).

## Discussion

Although it is well known that antiviral combination therapy is well tolerated and, in many cases, effective in the treatment of prolonged/relapsing COVID-19, herein we report a systematic use of such strategy also in the early phase of SARS-CoV-2 infection.

Herein we show that the early use of combination treatment with two antivirals targeting different viral proteins, together with the mAb sotrovimab in the early phase of COVID-19 in immunocompromised patients is effective and well tolerated. Indeed, there was a 100% clinical and virological response, whereas there was a lower response rate in patients treated in a later phase, when COVID-19 has already run a prolonged/relapsing course.

Our study is the first to evaluate the efficacy of early antiviral combination treatment in patients at risk of developing a prolonged/relapsing form of COVID-19. In fact, other studies have assessed similar combinations, but in patients with an established diagnosis of persistent SARS-CoV-2 infection. For example, Mikulska and colleagues reported a case series of 22 immunocompromised COVID-19 patients, 18 of which received a complete combination of two antivirals (remdesivir plus nirmatrelvir/ritonavir or molnupiravir) and one mAb and four received two antivirals after a median time of 42 (IQR 29–100) days from SARS-Cov-2 infection [6]. The authors obtained a response rate at days 14 and day 30, and at last follow-up of 75%, 73% and 82%, respectively. Moreover, there was a significantly higher response in patients receiving mAbs[6]. Additionally, Marangoni and colleagues recently described a case of clinical, radiological, and microbiological success in an immunosuppressed patient treated with a combination of nirmatrelvir/ritonavir (N/r) plus molnupiravir for prolonged symptomatic infection with SARS-CoV-2 [9].

It is still unclear whether the fact that patients in the late group had higher COVID-19 severity and need for oxygen and steroids is related to the delay in therapy administration or to factors that are independent of it. Nonetheless, the results of our study show that the use of early combination treatment with two antivirals targeting different viral proteins together with sotrovimab may cause a complete and long-lasting viral clearance and thus prevent the development of severe disease (need or increase of oxygen supplementation) in patients at risk of developing persistent COVID-19. The main limitation of the present study is the small number of observed patients, mostly due to the rarity of the studied condition. Another limitation is the short follow-up time for some patients; it is theoretically possible that some of the patients that we considered “cured” might develop a late relapse after the end of the follow-up period. Thus, prospective studies should include a longer follow-up and should compare traditional early single-drug therapy with an early combination regimen with different treatment durations in patients with COVID-19 and impairment of humoral immunity. However our results are encouraging.

## Data Availability

The data that support the findings of this study are available from the corresponding author (G.V.) upon reasonable request.
